# The Impact of Place of Residence on Antiretroviral Therapy Adherence: A Systematic Review and Meta-Analysis

**DOI:** 10.1155/arat/5757907

**Published:** 2025-02-22

**Authors:** Oluwaseun Abdulganiyu Badru, Joy Chioma Edeh, Rita Ifeyinwa Okonkwo, Luchuo Engelbert Bain, Oluwafemi Atanda Adeagbo

**Affiliations:** ^1^Department of Community and Behavioral Health, The University of Iowa, Iowa City, Iowa, USA; ^2^International Research Centre of Excellence, Institute of Human Virology, Abuja, Nigeria; ^3^Department of Epidemiology, The University of Iowa, Iowa City, Iowa, USA; ^4^Department of Psychology, Faculty of Humanities, University of Johannesburg, Auckland Park, Johannesburg, South Africa; ^5^International Programs Unit, APHRC, African Population Health Research Center, Nairobi, Kenya; ^6^Department of Sociology, University of Johannesburg, Johannesburg, South Africa

**Keywords:** antiretroviral therapy, ART adherence, HIV, meta-analysis, Nigeria, place of residence

## Abstract

**Objective:** There is evidence of geographical variation in HIV coverage and antiretroviral therapy (ART) adherence, and studies have investigated how the place of residence of people living with HIV (PLWH) influences ART adherence. Where people reside influences their access to health care. Studies on the influence of place of residence on ART adherence among PLWH in Nigeria have been reported in the literature. However, no review has synthesized these findings. Against this backdrop, this review seeks to determine whether adherence to ART differs by place of residence in Nigeria.

**Methods:** In May 2024, we searched four databases (CINAHL Plus, PubMed, Scopus, and Web of Science). Only empirical studies with a test of association between place of residence (i.e., urban and rural) and adherence to ART were included. We performed a fixed-effect meta-analysis with the meta package on R Studio Version 4.2.0.

**Results:** We included six of the 91 articles across the four databases. Most studies (*n* = 5) were conducted in the Southern region. The assessment of place of residence and adherence varies across the studies. We found that PLWH who reside in urban areas were 20% more likely to adhere to ART compared to those who live in rural areas (odds ratio: 1.20; 95% confidence interval: 1.01–1.43). Similarly, PLWH in the South-South region of Nigeria and reside in the urban areas were 1.27 (95% CI: 1.01–1.58) more likely to adhere to ART than those living in the rural areas. This observation was insignificantly true for the South-East region.

**Conclusion:** PLWH who reside in urban areas may better adhere to ART than their counterparts living in rural areas. Non-governmental organizations and government agencies working with PLWH should prioritize those living in rural areas because they are more likely to face greater barriers to adherence.

## 1. Introduction

More than 2 million people were living with HIV in Nigeria, making Nigeria the second highest HIV-burdened country globally as of 2023 [1]. Antiretroviral therapy (ART) adherence is crucial to the survival of people living with HIV (PLWH) globally, and there is evidence that some PLWH are not virally suppressed [2–4]. For instance, 93% of PLWH globally were virally suppressed as of 2023 [1], which is significantly higher than what is obtainable in Nigeria. Eight in ten (82%) PLWH in Nigeria were virally suppressed at the end of 2023, suggesting that ART medication adherence is suboptimal [1].

Several factors across different socioecological levels influence ART adherence, including HIV knowledge, poor socioeconomic status, substance use, HIV stigma and identity, and seropositive disclosure [5, 6]. Many studies on ART adherence often neglect an important determinant of health—place of residence (i.e., urban and rural areas) [7–10]. Where people live is arguably a stronger determinant of health, including the HIV care cascade, than many of the proximal social determinants mentioned earlier because where people reside influences their built environment and access to health infrastructure needed to enjoy better health [11–13].

The World Health Organization has emphasized the importance of where people live and work on health outcomes [14]. Non-HIV–related [15–18] and HIV-related studies [19–21] are beginning to single out place of residence and how they influence different health behaviors and health outcomes. Moreover, studies on ART adherence across Sub-Saharan Africa are beginning to beam light on the influence of place of residence on ART adherence [22, 23]. However, the relationship between place of residence and ART adherence is not linear, perhaps due to the lack of standard definitions for rural and urban between and within countries globally [13]. In Nigeria, population size is used to demarcate urban from rural areas, with places having < 20,000 inhabitants considered rural [24]. This definition of urban–rural area is more physical or “environmental” and may not account for other variations, including culture and economic activities, and this definition has been heavily criticized [13, 24, 25]. Moreover, most studies in Nigeria classify place of residence as urban or rural [26, 27]. As such, we adopted this classification for uniformity.

Some studies have reported that PLWH who reside in urban areas are more likely to adhere to ART medication [22, 23, 28]; others have found the reverse [29, 30]. One review attempted to pool studies that considered place of residence as a variable to determine whether urban dwellers adhere more to ART than rural dwellers in Ethiopia [31]. Another earlier review reported place of residence as a determinant of adherence among PLWH in sub-Saharan Africa [32]. However, we are unaware of a similar review in Nigeria—a country with unequal HIV prevalence across its six geopolitical zones and the second-highest HIV-burdened country globally [33]. Against this backdrop, this review seeks to determine whether adherence to ART differs by place of residence in Nigeria.

## 2. Materials and Methods

This systematic review and meta-analysis adhered to the Updated Systematic Review and Meta-Analysis Protocol (PRISMA-P) [34]. This review was registered on PROSPERO (CRD42022371965), and the protocol was published elsewhere [12].

### 2.1. Eligibility Criteria

The population, exposure, and outcome (PEO), as recommended by Moola et al. [35], was followed because it is more appropriate for reviews focused on the association between exposure and outcome. Studies were included if (1) they focused on PLWH in Nigeria, (2) measured place of residence and defined it as a rural, semi-urban, or urban area, (3) measured adherence to ART medication, and (4) demonstrated evidence of primary data collection (i.e., empirical studies). We excluded conference abstracts, commentaries, and letters to editors.

### 2.2. Database and Search Strategy

#### 2.2.1. Database

Four databases (CINAHL Plus, PubMed, Scopus, and Web of Science) were searched for relevant articles in May 2024 without a date or language filter. Google Scholar and a reference list of the included articles were also searched for more relevant articles. The search strategy consisted of relevant keywords and Medical Subject Heading (MeSH) terms (“HIV”[Mesh] OR “persons living with HIV”) AND (Antiretroviral therapy OR ART) AND (adherence OR compliance) AND (“place of residence” OR urban OR rural) AND (Nigeria). These keywords, where necessary, were truncated to explode the search and put in parentheses to improve search sensitivity in PubMed (Additional file 1), Scopus (Additional file 2), Web of Science (Additional file 3), and CINAHL Plus (Additional file 4).

#### 2.2.2. Search Strategy

One reviewer (O.A.B.) performed the systematic search and exported the articles to Rayyan for screening [36]. One reviewer (O.A.B.) performed deduplication. Two reviewers (O.A.B. and J.C.E.) independently screened the title and abstract and resolved conflicts through dialogue. Subsequently, both reviewers (O.A.B. and J.C.E.) independently performed the full-text screening and resolved discrepancies through discussion.

### 2.3. Data Extraction

O.A.B. and J.C.E. extracted data from the included studies individually into Microsoft Word, compared findings, and resolved discrepancies through dialogue. The following details were extracted from the included studies: author and publication year, study design, state where the study was conducted within Nigeria, ART adherence measurement (validated or unvalidated tool), participant characteristics (age and gender), sample size, and reported ART medication adherence proportion.

### 2.4. Quality Assessment

O.A.B. and J.C.E. independently evaluated the methodological rigor of the included studies using the Joanna Briggs Institute appraisal tool for prevalence studies [37]. The tool has nine items measured with three options: “yes,” “no,” and “unclear.” One point was allocated per checklist, and the scores were converted to percentages; only studies with < 50% were considered to have a low risk of bias [38].

### 2.5. Data Analysis

R Studio Version 4.2.0 was utilized to perform all data analysis. We used the meta package to run the meta-analysis. How ART adherence differed between urban and rural settings was summarized with odds ratio. Higgins and Thompson's *I*^2^ and Cochrane's *Q* test were used to ascertain heterogeneity. Low, moderate, and high heterogeneity was defined as *I*^2^ of ≤ 25%, ≤ 50%, and ≥ 75%, respectively [39]. A fixed-effect model was utilized because there was zero heterogeneity between the studies. Also, we performed a random-effect model to account for possible place of residence variability and to assess whether the odds ratio and heterogeneity will differ by methods (Additional file 5). Although not indicated since there was no heterogeneity between the studies, we performed subgroup analyses by region within Nigeria (South-East, South-South, and North Central) because of the regional difference in HIV burden. We assessed publication bias with visual funnel plot inspection and Egger's test [40]. Study characteristics were narratively synthesized.

## 3. Results

**Figure 1:**
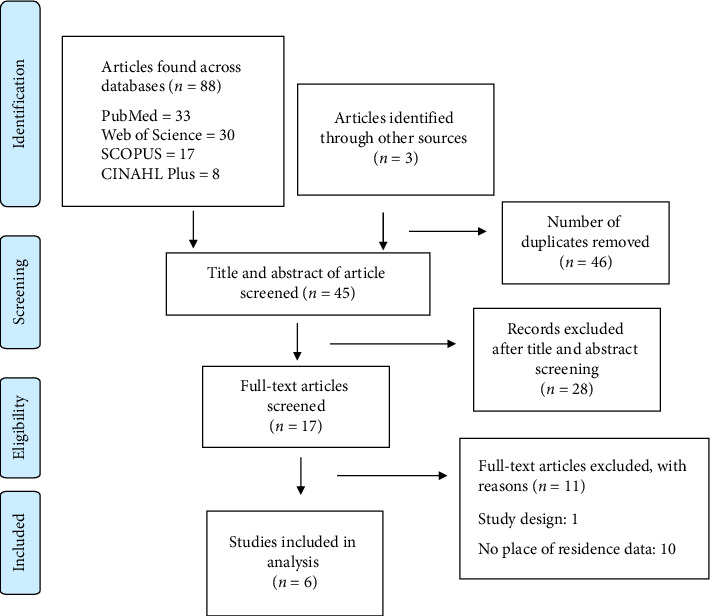
Search strategy flowchart.

We found 91 articles across the four databases (PubMed = 33, Web of Science = 30, Scopus = 17, and CINAHL Plus = 8) and hand-searched records (*n* = 3). Forty-six of the 91 articles were duplicates and, therefore, excluded, leaving 45 articles for title and abstract screening. Subsequently, 28 articles were deleted, leaving 17 articles for full-text screening. Additional exclusions were studies that did not contain adherence and/or place of residence data (*n* = 10) and one qualitative study (*n* = 1). Six studies were analyzed in this review (Figure 1).

### 3.1. Study Description

As shown in Table 1, six studies were conducted among 3765 PLWH in four of Nigeria's 36 states and the Federal Capital Territory. Two studies each were conducted in Cross River [30, 41] and Enugu [23, 42], while one study each was conducted in Anambra [43] and Plateau State [44]. Classifying these states into the six geopolitical zones in Nigeria, one study was conducted in the North Central [44], two in the South-South [30, 41], and three studies in South Eastern region [23, 42, 43].

Regarding study setting, two studies utilized one health facility [42, 43], one study utilized two facilities [41], and two studies collected data from four facilities [30, 44], while Chime, Ndibuagu, and Orji [23] surveyed PLWH across eight facilities. All the studies adopted probability random sampling. Specifically, two studies each sampled participants with simple random sampling [42, 43], systematic random sampling [23, 41], and multistage sampling [30, 44].

All the studies operationalized adherence differently; however, most (*n* = 4) leveraged single-item adherence questions [23, 30, 42, 44], while two studies utilized the Morisky Medication Adherence Scale (MMAS) [41, 43]. Among the studies that assessed ART adherence with a single item, one study each asked participants to recall missed doses in the past four days [42], seven days [30], 28 days [23], and 30 days [44]. One of the two studies that leveraged MMAS used the four-item version [43], while the other study employed the eight-item version [41]. None of the studies operationalized the place of residence in their methodology nor utilized a theory, framework, or model.

### 3.2. Adherence by Place of Residence

**Figure 2:**
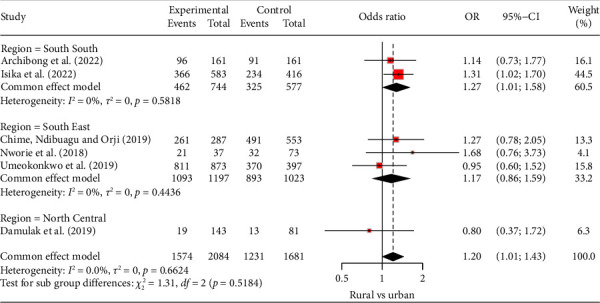
Adherence by place of residence.

**Figure 3:**
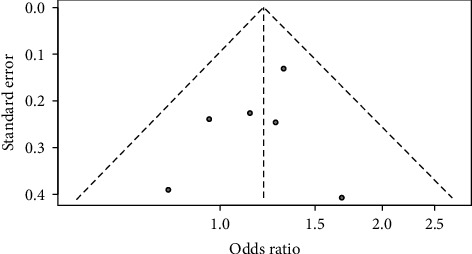
Funnel plot of publication bias.

Four of the six studies showed that adherence was better in urban areas than in rural areas (Figure 2). Specifically, these studies show that the odds of better ART adherence among urban dwellers ranged from 14% to 68% compared to rural dwellers. Overall, PLWH who reside in urban areas were 20% more likely to adhere to ART compared to those who live in rural areas (odds ratio [OR]: 1.20, 95% confidence interval [CI]: 1.01–1.43); there was no evidence of heterogeneity (*I*^2^ = 0%; *p* = 0.66) between the studies as shown in the fixed-effect model. Moreover, there was no significant change in effect size with sensitivity analysis. Furthermore, we explored places of residence in different geopolitical zones in Nigeria and found that PLWH in the South-South region of Nigeria and residing in the urban areas were 1.27 (95% CI: 1.01–1.58; *I*^2^ = 0%; *p* = 0.58) more likely to adhere to ART than those residing in the rural areas. A similar finding was observed in the South-East, but not to a significant level (OR: 1.17, 95% CI: 0.86–1.59; *I*^2^ = 0%; *p* = 0.44) (Figure 2). We repeated this analysis using a random-effect model to account for possible variability of place of residence and found a similar odds ratio and heterogeneity (see Supporting 5). The visually inspected funnel plot and Egger's test (*t* = 1.37, *p* = 0.244) show no evidence of publication bias (Figure 3).

## 4. Discussion

This review investigated whether ART adherence differs by where people live in Nigeria. We found only six ART adherence-related studies that considered place of residence of PLWH; five were conducted in the Southern part of Nigeria. This calls for a need to include place of residence as a variable in ART adherence studies. Of note, only three of the six studies mentioned place of residence in their methodology [41, 42, 44], and none of them operationalized place of residence. We also observed non-operationalization of place of residence in a previous similar systematic review [31]. None of the seven studies found by Fite [31] operationalized place of residence [31]. This perhaps indicates that non-operationalization of place of residence appears to be common in ART adherence-related research.

How place of residence is captured is important and should be documented. For instance, asking PLWH to classify their residence into urban and rural may be erroneous because they may not correctly categorize where they live, leading to information and reporting bias, misrepresentation of the variable, and distortion of the association with ART adherence [45]. Several ways have been proposed to capture place of residence [13, 46]. Wineman, Alia, and Anderson [46] proposed seven ways to operationalize place of residence, including administrative definition (country's official designation of rural and urban areas with zip code), and population density of 500 persons/km^2^ as urban area. Context matters in classifying rural–urban areas, and there are no universal definitions of rural and urban areas; description varies considerably between and within countries [13, 47]. Nevertheless, future studies interested in the place of residence are encouraged to operationalize it clearly so that other authors can reproduce their studies [13].

All the studies assessed ART adherence with either the Morisky tool or a single-item question (focused on the number of pills used in a specific period). These techniques are susceptible to recall bias and social desirability, making them not the best strategy to assess ART adherence; viral load suppression remains the gold standard for monitoring and confirming ART adherence [48]. To observe a more reliable adherence measure, researchers without funding for viral load assessment and who do not have access to patient records can ensure that research assistants are adequately trained to collect accurate adherence data, preferably with validated multi-item tools such as the Morisky 8-item tool [49]. Multiple-item tools offer better construct validity and reliability than single-item adherence tools [50].

We found that PLWH who reside in urban areas were significantly more likely to adhere to ART medication than those who live in rural areas, including in all the regions. This finding is similar to that of Fite [31] who found that residing in urban areas was associated with better adherence to ART among PLWH in Ethiopia. However, another meta-analysis among PLWH in Sub-Saharan Africa found that those who reside in the urban areas were three-fold less likely (OR: 3.32, 95% CI: 2.26–4.87, *I*^2^ = 0%) to adhere to ART [32].

There are several plausible reasons for our finding. Urban regions have more healthcare facilities that provide HIV care continuum than rural areas, with increased access to medication refills for urban dwellers. Although multimonth ART dispensing increases access to ART medication for rural dwellers [51], the proximity to health facilities, especially facilities in the urban regions for medication refills when needed, is a threat to ART adherence in Nigeria [52]. As such, PLWH in rural areas are more likely to miss appointments and not adhere to their medication [31]. Another explanation is that most PLWH in rural areas may be low-income earners, which may affect their ability to convey themselves to the health facilities for refill and follow-up [53].

### 4.1. Limitations

The findings should be interpreted in the context of several limitations. The studies assessed adherence mainly through self-reports, which may be less accurate and impact our findings. Moreover, self-reports are prone to recall bias, social desirability, and overestimating adherence, which can limit the understanding of adherence and reduce the validity of our findings. Furthermore, the included studies did not report how the place of residence was operationalized, and we assume it may have been captured as a self-report with the risk of information and report bias. This lack of consistency in the definition of place of residence has implications for our findings. The respondents may have inaccurately classified their residence or exhibit social desirability. Furthermore, the conclusion that persons who reside in urban areas have better adherence than those who live in rural cannot be conclusive. Moreover, the findings cannot be generalized within or outside Nigeria. Also, only three of the six geopolitical zones in Nigeria were represented in this study (South-East, South-South, and North Central); therefore, the findings may not apply to the non-represented regions (South West, North West, and North East). Moreover, the studies found for these regions are too few to draw conclusions. Nonetheless, this review is the first to assess the influence of place of residence on ART adherence in Nigeria.

## 5. Conclusions

PLWH residents in urban areas showed better ART adherence than their counterparts living in rural areas, but more studies are needed to substantiate this finding. Therefore, we recommend that the findings be interpreted with caution. More studies should include place of residence as a variable in their research and operationalize it clearly. Researchers should also consider the fact that rural areas and slums exist within urban areas and vice versa. Although this makes place of residence operationalization more complex, it allows for more realistic and accurate data. Future studies should provide operational definitions for place of residence to allow for contextual interpretation of reported findings. Moreover, future adherence studies should capture adherence with viral load suppression because it is the gold standard for ART adherence measurement. Non-governmental organizations and government agencies working among PLWH should not neglect PLWH in the rural region, as there is evidence that they may be lagging in ART adherence. Rural areas should be prioritized for HIV advocacy, mainly because they may have limited structural, material, and human resources than the urban areas, which may negatively influence the HIV care continuum of PLWH in the rural areas.

## Figures and Tables

**Table 1:** Characteristics of the included studies.

SN	Author	Study design/state	Study setting/sampling	Adherence measurement	Participant's characteristics	Sample size	Adherence by region	Risk
1	Archibong et al. [41]	Cross-sectional/Cross River	2 health facilities/systematic sampling technique	Morisky 8-item Medication Adherence Questionnaire: ≥ 95%	ART for at least 6 months	322 (161 per site)	95% pill use	Low
					Mean age ± SD		Adherence: 187/322 = 58.1%	
					Urban: 34.96 ± 7.11		Urban: 96/161 = 59.6%	
					Rural: 34.07 ± 7.00		Rural: 91/161 = 56.5%	
					Total: 34.52 ± 7.00		Self-reporting adherence	
					Gender (male)		Overall: 202/322 = 62.7%	
					Total: 153 (47.5%)		Urban: 107/161 = 66.5%	
					Urban: 76 (47.2%)		Rural: 95/161 = 59.0%	
					Rural: 77 (47.8%)		Adherence on pharmacy records	
					Gender (female)		Overall: 186/322 = 57.7%	
					Total: 169 (52.5%)		Urban: 106/161 = 65.8%	
					Urban: 85 (52.8%)		Rural: 80/161 = 49.7%	
					Rural: 84 (52.2%)			

2	Chime, Ndibuagu, and Orji [23]	Cross-sectional/Enugu	8 health facilities/systematic sampling technique	Single item on pills used in the past 28 days: ≥ 95%	ART for at least a year Mean age ± SD 38.5 ± 9.8 Gender Male: 199 (23.7%) Female: 641 (76.3%)	840	Total adherence: 752/840 = 89.5%	Low
							Urban: 261/287 = 90.9%	
							Rural: 491/553 = 88.8%	

3	Isika et al. [30]	Cross-sectional/Cross River	4 health facilities/multistage sampling technique: (Stage 1: SRS; Stage 2: SRS; Stage 3: systematic random sampling)	Single item on pills used in the past 7 days: ≥ 95%	ART for at least 3 months	999	Total adherence: 600/999 = 60.1% Urban: 366/ 583 = 62.8% Rural: 234/416 = 56.3%	Low
					Mean age ± SD			
					43.7 ± 11.1			
					Gender			
					Male: 295 (29.5%)			
					Female: 704 (70.5%)			

4	Damulak et al. [42]	Cross-sectional/Plateau	1 health facility/SRS	Single item on pills used in the past 4 days: ≥ 95%	ART for at least 6 months (and viral load > 1000 copies/mL) Modal age 41–50: 85 (37.9%) Gender Male: 90 (40.2%) Female: 134 (59.8%)	224	Total adherence:	Low
							32/224 = 14.3%	
							Urban: 19/143 = 13.3%	
							Rural: 13/81 = 16.1%	

5	Nworie et al. [43]	Cross-sectional/Enugu	1 health facility/SRS	Morisky Medication Adherence Scale (MMAS-4): score < 0.95 from 4	ART duration for inclusion not stated	110	Total adherence: 53/110 = 48.2% Urban: 21/37 = 56.8% Rural: 32/73 = 43.8%	Low
					Mean age ± SD			
					39.7 ± 11.39			
					Gender			
					Male: 27 (24.5%)			
					Female: 83 (75.5%)			

6	Umeokonkwo et al. [44]	Cross-sectional/Anambra	4 health facilities/multistage sampling technique	Single item on pills used in the past 30 days: ≥ 95%	ART for at least a year	1270 (635 each for public and private health facilities)	Total adherence: 1181/1270 = 93.0% Urban: 811/873 = 92.9% Rural: 370/397 = 93.2%	Low
					Modal age			
					≥ 35: 869 (68.4%)			
					Gender (*n* = 1260)			
					Male: 616 (48.9%)			
					Female: 644 (51.1%)			

Abbreviations: SD, standard deviation; SRS, simple random sampling.

## Data Availability

The data that support the findings of this study are available on request from the corresponding author. The data are not publicly available due to privacy or ethical restrictions.

## Supporting Information

Supporting 1. Additional File 1. File name: Additional file 1. Title: search strategy for PubMed. Description of data: The material contains specific key terms for PubMed database search.

Supporting 2. Additional File 2. File name: Additional file 2. Title: search strategy for Scopus. Description of data: the material contains specific key terms for Scopus database search.

Supporting 3. Additional File 3. File name: Additional file 3. Title: search strategy for Web of Science. Description of data: the material contains specific key terms for Web of Science database search.

Supporting 4. Additional File 4. File name: Additional file 4. Title: search strategy for CINAHL Plus. Description of data: the material contains specific key terms for CINAHL Plus database search.

Supporting 5. Additional File 5. File name: Additional file 5. Title: random-effect meta-analysis. Description of data: the random-effect analysis was conducted to see if there is a difference compared to fixed-effect analysis.
