# Supplementary material for: The Impact of Place of Residence on Antiretroviral Therapy Adherence: A Systematic Review and Meta-Analysis
Source: AIDS Res Treat. 2025 Feb 22;2025:5757907. doi: 10.1155/arat/5757907 (PMC11871977; doi:10.1155/arat/5757907)
Supplement: Supporting Information — Additional supporting information can be found online in the Supporting Information section. [file 5757907.f1.zip › Additional file 1.docx]

**SEARCH STRATEGY**

**Additional file 1: Search strategy for PubMed**

| SEARCH | ALL TERMS | HIT |
| --- | --- | --- |
| #1 | **"HIV"[Mesh] OR "HIV Long-Term Survivors"[Mesh] OR HIV OR "human immunodeficiency virus" OR "acquired immunodeficiency syndrome" OR AIDS OR HIV/AIDS OR "people living with HIV" OR "persons living with HIV" OR "people living with AIDS" OR "persons living with AIDS" OR "people living with HIV/AIDS" OR "persons living with HIV/AIDS" OR "HIV-infected person*"** | 563,069 |
| #2 | **"Antiretroviral Therapy, Highly Active"[Mesh] OR "antiretroviral therapy" OR "Highly Active Antiretroviral Therapy" OR ART OR cART OR HAART** | 275,715 |
| #3 | **"Medication Adherence"[Mesh] OR adherence OR compliance OR "non-compliance" OR "medication compliance" OR "Treatment Adherence" OR "Failure to Adhere"** | 501,899 |
| #4 | **"Residence Characteristics"[Mesh] OR "place of residence" OR "area of residence" OR urban OR "urban area*" OR "urban setting*" OR rural OR "rural area*" OR "rural setting*"** | 578,745 |
| #5 | **"Nigeria"[Mesh] OR Nigeria OR Abuja OR Abia OR Adamawa OR "Akwa Ibom" OR Anambra OR Bauchi OR Bayelsa OR Benue OR Borno OR "Cross River" OR Delta OR Ebonyi OR Edo OR Ekiti OR Enugu OR Gombe OR Imo OR Jigawa OR Kaduna OR Kano OR Katsina OR Kebbi OR Kogi OR Kwara OR Lagos OR Nasarawa OR Niger OR Ogun OR Ondo OR Osun OR Oyo OR Plateau OR Rivers OR Sokoto OR Taraba OR Yobe OR Zamfara** | 500,372 |
|  | **(((("HIV"[Mesh] OR "HIV Long-Term Survivors"[Mesh] OR HIV OR "human immunodeficiency virus" OR "acquired immunodeficiency syndrome" OR AIDS OR HIV/AIDS OR "people living with HIV" OR "persons living with HIV" OR "people living with AIDS" OR "persons living with AIDS" OR "people living with HIV/AIDS" OR "persons living with HIV/AIDS" OR "HIV-infected person*") AND ("Antiretroviral Therapy, Highly Active"[Mesh] OR "antiretroviral therapy" OR "Highly Active Antiretroviral Therapy" OR ART OR cART OR HAART)) AND ("Medication Adherence"[Mesh] OR adherence OR compliance OR "non-compliance" OR "medication compliance" OR "Treatment Adherence" OR "Failure to Adhere")) AND ("Residence Characteristics"[Mesh] OR "place of residence" OR "area of residence" OR urban OR "urban area*" OR "urban setting*" OR rural OR "rural area*" OR "rural setting*")) AND ("Nigeria"[Mesh] OR Nigeria OR Abuja OR Abia OR Adamawa OR "Akwa Ibom" OR Anambra OR Bauchi OR Bayelsa OR Benue OR Borno OR "Cross River" OR Delta OR Ebonyi OR Edo OR Ekiti OR Enugu OR Gombe OR Imo OR Jigawa OR Kaduna OR Kano OR Katsina OR Kebbi OR Kogi OR Kwara OR Lagos OR Nasarawa OR Niger OR Ogun OR Ondo OR Osun OR Oyo OR Plateau OR Rivers OR Sokoto OR Taraba OR Yobe OR Zamfara)** | 33 |
