# Supplementary figures and images for: The Impact of Place of Residence on Antiretroviral Therapy Adherence: A Systematic Review and Meta-Analysis
Source: AIDS Res Treat. 2025 Feb 22;2025:5757907. doi: 10.1155/arat/5757907 (PMC11871977; doi:10.1155/arat/5757907)

**
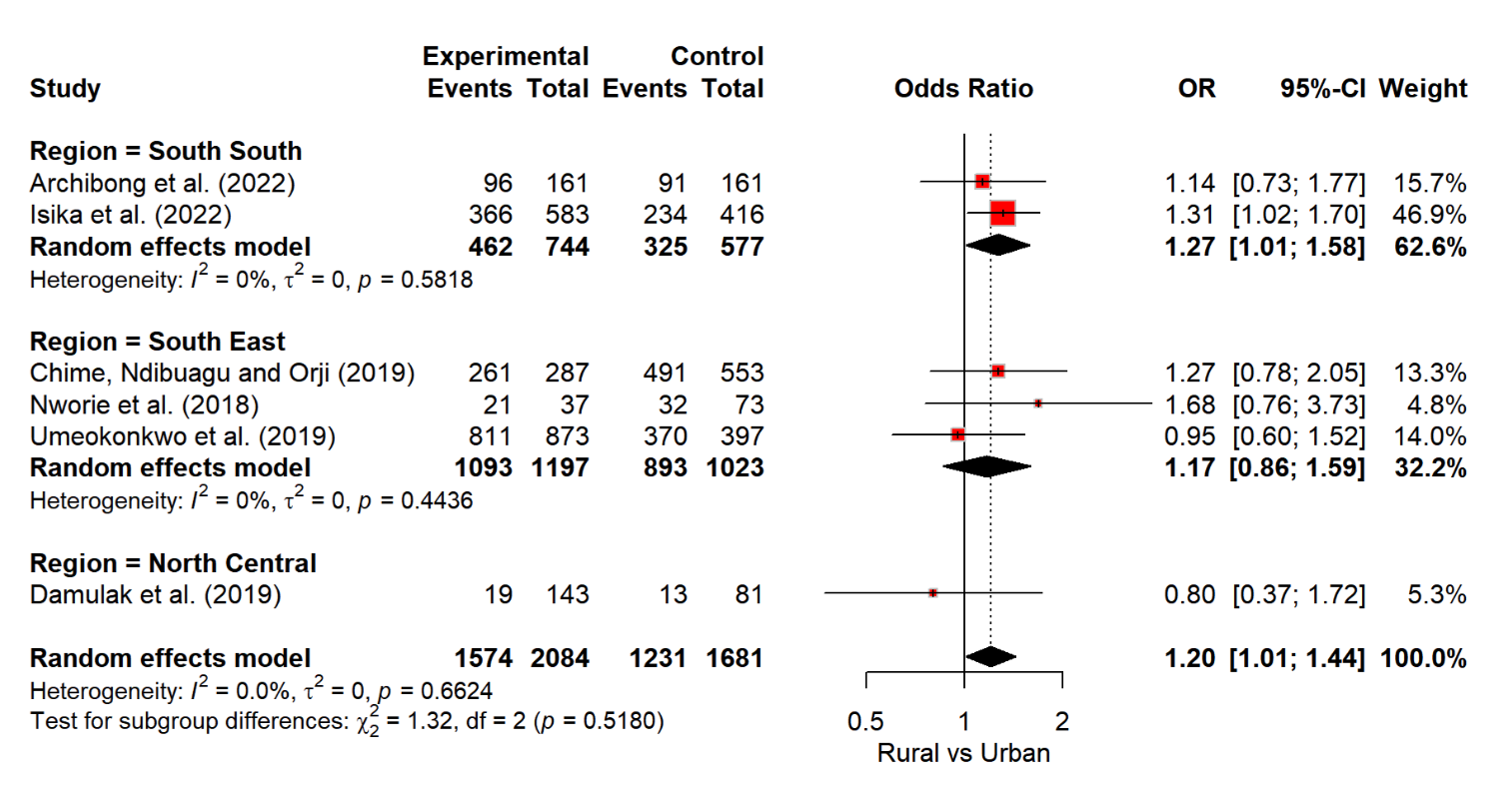
**

**Additional file 5: Random effect meta-analysis**

Supplement: Supporting Information — Additional supporting information can be found online in the Supporting Information section. [file 5757907.f1.zip › Additional file 5.docx]

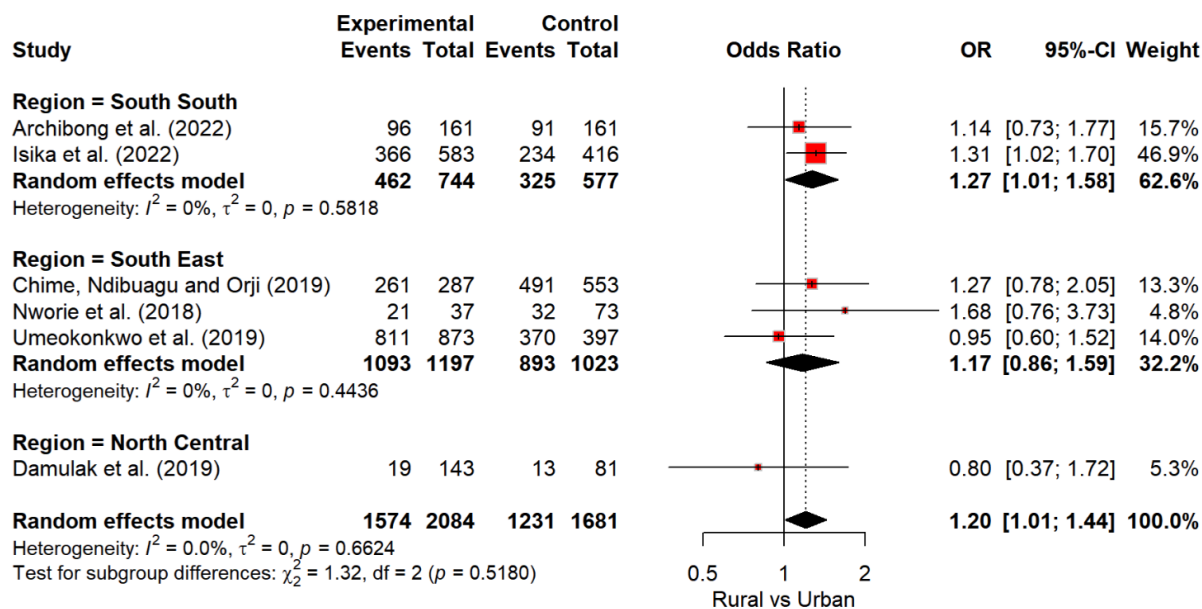

## Additional file 5: Random effect meta-analysis

Supplement: Supporting Information — Additional supporting information can be found online in the Supporting Information section. [file 5757907.f1.zip › Additional file 5.pdf]
